# Interrelation of the spatial and genetic structure of tick‐borne encephalitis virus, its reservoir host (*Myodes glareolus*), and its vector (*Ixodes ricinus*) in a natural focus area

**DOI:** 10.1002/ece3.70163

**Published:** 2024-08-19

**Authors:** Lea Kauer, Gerhard Dobler, Hannah M. Schmuck, Lidia Chitimia‐Dobler, Martin Pfeffer, Ralph Kühn

**Affiliations:** ^1^ Molecular Zoology, Department of Zoology, TUM School of Life Sciences Technical University of Munich Freising Germany; ^2^ Bundeswehr Institute of Microbiology Munich Germany; ^3^ Institute of Animal Hygiene and Veterinary Public Health University of Leipzig Leipzig Germany; ^4^ Department of Fish, Wildlife and Conservation Ecology New Mexico State University Las Cruces New Mexico USA

**Keywords:** bank voles, habitat corridors, host–parasite ecology, landscape genetics, tick‐borne disease, zoonosis

## Abstract

Tick‐borne encephalitis (TBE) virus is considered the medically most important arthropod‐borne virus in Europe. Although TBE is endemic throughout central Europe, ticks and rodents determine its maintenance in small, difficult‐to‐assess, natural foci. We investigated the interrelation between the population genetics of the main TBE virus (TBEV) vector tick (*Ixodes ricinus*), the most important reservoir host (*Myodes glareolus*, syn. *Clethrionomys glareolus*), and TBEV. Rodents and ticks were sampled on 15 sites within an exploratory study area, which has been screened regularly for TBEV occurrence in ticks for more than 10 years. On all 15 sites, ticks and bank voles were sampled, screened for TBEV presence via serology and RT‐PCR, and genetically examined. Moreover, TBEV isolates derived from these analyses were sequenced. In long‐term TBEV foci bank vole populations show extraordinary genetic constitutions, leading to a particular population structure, whereas ticks revealed a panmictic genetic structure overall sampling sites. Landscape genetics and habitat connectivity modeling (analysis of isolation by resistance) showed no landscape‐related barriers explaining the genetic structure of the bank vole populations. The results suggest that bank voles do not simply serve as TBEV reservoirs, but their genetic composition appears to have a significant influence on establishing and maintaining long‐term natural TBEV foci, whereas the genetic structure of TBEV's main vector *I. ricinus* does not play an important role in the sustainability of long‐term TBEV foci. A thorough investigation of how and to which extent TBEV and *M. glareolus* genetics are associated is needed to further unravel the underlying mechanisms.

## Graphical abstract

Tick‐borne encephalitis is endemic throughout central Europe, ticks and rodents determine its maintenance in small, difficult‐to‐assess, natural foci. In long‐term TBEV foci bank vole populations show extraordinary genetic constitutions, leading to a particular population structure, whereas ticks revealed a panmictic genetic structure over all sampling sites. The results suggest that bank voles do not simply serve as TBEV reservoirs, but their genetic composition has a significant influence on establishing and maintaining long‐term natural TBEV foci, whereas the genetic structure of TBEV's main vector *Ixodes ricinus* does not play an important role in the sustainability of long‐term TBEV foci.

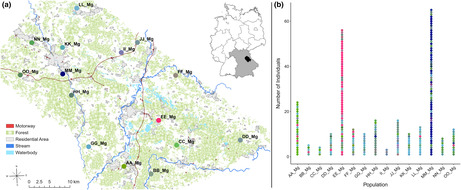

## 1 INTRODUCTION

Tick‐borne encephalitis (TBE), a disease‐causing potentially severe neurological symptoms in patients, is endemic throughout many European countries, with usually higher incidences in the Baltic and central European countries and an annual number of TBE cases fluctuating between 2000 and 4000 cases in the European Union (Beauté et al., 2018; Lindquist & Vapalahti, 2008). TBE is caused by the tick‐borne encephalitis virus (TBEV), a zoonotic flavivirus that is considered the medically most crucial arthropod‐borne virus (arbovirus) in Europe (Randolph, 2011; Süss, 2011; Tonteri et al., 2013).

In 2020, TBE cases in Germany reached an all‐time high with 706 confirmed cases, representing an increase of cases of 59% compared with 2019, with a hospitalization rate of 85% of patients (European Centre for Disease Prevention and Control, 2022). The virus is maintained in a transmission cycle involving ticks and small mammals within small, locally restricted, so‐called microfoci (Borde et al., 2022). In Europe, *Ixodes ricinus* ticks function as the main arthropod vector for TBEV, whereas bank voles (*Myodes glareolus*, syn. *Clethrionomys glareolus*) serve as a main reservoir host for TBEV (Knap et al., 2012; Süss, 2011). Additionally, rodents are hosts for the juvenile stages of *I. ricinus* (Mihalca & Sándor, 2013). Several ways of transmission and maintenance of TBEV are known (Chitimia‐Dobler et al. (2019). Ticks become infected by feeding on a viremic host (Mansfield et al., 2009) or co‐feeding on a non‐viremic host (Labuda et al., 1997; Randolph, 2011) and perpetuate the infection transstadially (Karbowiak & Biernat, 2016) or rarely transovarially (Danielová et al., 2002). Therefore, all stages of ticks can become infected with the virus, and all hematophagous stages can also transmit the virus to vertebrate hosts (Grzybek et al., 2018). Furthermore, rodents play an essential role in maintaining TBEV in nature by carrying persistent latent infections (Tonteri et al., 2011; Zöldi et al., 2015). Many studies underline the crucial role *I. ricinus* ticks and rodents, especially *M. glareolus* (Zöldi et al., 2015), play in maintaining and transmitting TBEV.

Although many different TBEV strains have been genetically characterized (Sukhorukov et al., 2023), little is known about the genetic structure of the vectors (*I. ricinus*) and reservoir hosts (*M. glareolus*) of TBEV in natural TBEV foci or the spatial genetic interrelation between vector, reservoir, and TBE virus. Our study integrates population genetic analyses of both the vector, *I. ricinus*, and the reservoir host, *M. glareolus*, shedding light on the genetic dynamics within these populations and their potential implications for establishing and maintaining TBEV natural foci. Moreover, we incorporate genetic data of TBEV strains isolated from our study plots. This holistic approach is novel and critical for understanding the intricate interplay between the genetic makeup of vectors, reservoirs, and the pathogen itself, which has not been comprehensively explored in previous research. In this study, we investigate the genetic structure of the TBEV vector species *I. ricinus* and the TBEV reservoir species *M. glareolus* on 15 sampling sites, including known TBEV foci and sites with no information about TBEV occurrence, and data regarding the genetic structure of TBEV strains isolated from six sites. Combing genetic data of vector, reservoir, and pathogen with habitat suitability and corridor analysis of the reservoir species bank vole, we aim to estimate the role the genetic composition of vectors (*I. ricinus)* and host (*M. glareolus*) may play in the distribution and transmission of TBE virus.

## 2 MATERIALS AND METHODS

### 2.1 Sampling of *Myodes glareolus* and *Ixodes ricinus*

Sampling of rodents and ticks took place on 15 sites within an established exploratory study area in southern Germany, which has been screened regularly for TBEV occurrence in ticks for more than 10 years (Brugger et al., 2018). In the course of these evaluations, two plots, EE and MM have been found to be well‐established TBEV natural foci. Rodents were trapped from March to October 2019 (see Table 1 and Figure 2) using Sherman Traps. Permission was granted through the district government of Upper Palatinate (ROP‐SG55.1‐8646.4‐1‐125‐2). The traps were placed along the ecotone with an approximate distance of 5 m between traps plus, if the site allowed for entering the forest, approximately 5 m inside the forest in a 5 m distance. Five sampling nights took place on each site, with at least 5 days between each sampling event. Bank voles were anesthetized using Isoflurane, euthanized by cervical dislocation, immediately transferred to dry ice, and stored at −80°C until further processing. All animal handling was performed in accordance with Directive 2010/63/EU. On sites EE and MM, additional bank vole sampling was conducted by Brandenburg et al. (2023) in 2019.

**TABLE 1:** Geographic location of the 15 sampling sites and demographic parameters (*Myodes glareolus*) and tick (*Ixodes ricinus*) populations.

Research area			*Myodes glareolus*				*Ixodes ricinus*					
Plot ID^a^	Longitude	Latitude	*N* ^b^	Abund.^c^/100 nights	Male	Female	*N*	Abund.^c^/100 m^2^	Larvae	Nymphs	Adults male	Adults female
AA	49.19892	12.07769	24	14.1	19	5	67	10.2	6	50	8	3
BB	49.17970	12.16772	5	2.7	4	1	331	42.4	151	163	7	10
CC	49.24109	12.25734	4	5.2	4	0	82	10.9	37	39	4	2
DD	49.24593	12.47169	10	3.6	3	7	140	16.7	103	27	5	5
EE	49.29750	12.20065	18	9.3	15	3	191	45.9	97	78	7	9
FF	49.39534	12.26432	12	6.7	8	4	276	30.7	183	70	15	8
GG	49.24542	11.96195	10	5.4	5	5	76	9.1	19	38	13	6
HH	49.36088	11.91060	16	9.4	7	9	31	6.5	3	22	3	3
II	49.45090	12.08438	3	1.6	2	1	131	20.5	71	50	4	6
JJ	49.47168	12.14033	16	9.0	9	7	250	33.3	192	47	8	3
KK	49.46723	11.88715	10	8.1	5	5	33	15.7	10	17	2	4
LL	49.55293	11.94014	13	5.4	9	4	50	8.3	15	29	1	5
MM	49.40849	11.88422	36	13.9	26	10	1425	129.2	972	397	29	27
NN	49.48038	11.78416	8	5.0	5	3	465	64.6	108	261	52	44
OO	49.40969	11.74122	12	7.0	6	6	76	19.5	13	60	3	0
Total			197	7.1	127	70	3624	463.4	1980	1348	161	135

^a^ Plot identifier.

^b^ Number of animals

^c^ Abundance.

Ticks were sampled from June to October 2017–2019 using the flagging method with a 1m^2^ cotton cloth. Subsamples of ticks were taken from vegetation strips of 10 m with 10 m in between the strips. After each 10 m strip, the ticks were removed from the cloth, stored in a tube, and taxonomically classified. Larvae, nymphs, and adult ticks were selected for analysis proportionally to occurring life stages. The ticks were stored in RNAlater at −20°C until further processing.

### 2.2 TBE virus detection

RNA extraction of TBEV from bank voles was performed on brain tissue, which has been shown to be an ideally suitable organ for the detection of TBEV RNA (Achazi et al., 2011; Kovac & Moritsch, 1959; Michelitsch et al., 2021; Tonteri et al., 2011). *I. ricinus* ticks were processed in pools of 10 nymphs or five adults per pool. The extracted nucleic acid from bank vole and tick samples was tested for TBEV RNA using the RT‐PCR as described by Schwaiger and Cassinotti (2003) in order to detect the presence of TBEV RNA. Virus isolation from brains and other organs of rodents was conducted as described in Boelke et al. (2019) except that 10% of organ homogenates were used as inoculum in cell culture. Additionally, all bank voles were screened serologically for the presence of TBEV antibodies via Indirect Immunofluorescence Assay (IIFA) (FSME‐Viren (TBEV), Euroimmun AG, Luebeck, Germany), see Brandenburg et al. (2023) for detailed information.

### 2.3 Genetic analysis of *Myodes glareolus*, *Ixodes ricinus*, and TBE virus

DNA extraction was performed on bank vole tail‐tissue samples and whole ticks using phenol–chloroform–Isopropanol extraction (Hogan et al., 1986). For genetic analysis, we applied a set of 12 microsatellite loci for *M. glareolus* (CG13G2, CG5F6, CG16E2, CG17E9, CG7C9, CG15F7, CG12B9, CG13F9, CG5G56, CG12A7 (Rikalainen et al., 2008) and MSCg‐15 (Gockel et al., 1997)) as well as a set of 12 microsatellite loci for *I. ricinus* (IR27, IR32, IR39, IR8, (Delaye et al., 1998) IRic05, IRic08, IRic11, (Kempf et al., 2011) IRic09, IRic13 (Noel et al., 2012) IRN‐3, IRN‐7, and IRN‐14 (Roeed et al., 2006)). Multiplex PCR was performed in a total volume of 15 μL containing a maximum of 24 ng of genomic DNA using the QIAGEN Multiplex PCR Kit (QIAGEN). Primer concentration varied between 0.15 and 0.25 μM in the bank vole multiplex system and 0.06 and 0.21 μM in the tick multiplex system (see Appendix S1). The bank vole multiplex protocol describes an initial denaturation at 95°C for 5 min, 35 cycles of 94°C for 30 s, 55°C respectively, 60°C for 90 s, 72°C for 30 s, and a final extension at 68°C for 10 min. The tick multiplex protocol describes an initial denaturation at 95°C for 5 min, 35 cycles of 95°C for 30 s, 58°C for 90 s, 72°C for 30 s, and a final extension at 68°C for 10 min. Fragment sizes were determined by electrophoresis on 4.5% (w/v) denaturing 19:1 acrylamide:bisacrylamide gels on the ABI Prism™ 377 sequencer, using the GeneScan 2.0 software and a ROX‐labeled commercial size standard as an internal standard (Applied Biosystems).

RT‐PCR‐positive tissue was used to isolate and molecularly characterize the respective TBEV strain, according to Kupča et al. (2010). For detailed information regarding RNA extraction and virus isolation, see Chitimia‐Dobler et al. (2019. Envelope (E) gene sequencing was performed as described previously in Weidmann et al. (2011).

### 2.4 Statistical analysis

We arranged microsatellite data with the Excel Microsatellite Tool Kit 3.1.1 (Park, 2001) and converted data into the favored file types. With FSTAT v. 2.9.3 (Goudet, 2001) allele frequencies, average allele numbers per locus (A), allelic richness (*A*_R_), expected and observed heterozygosities (*H*_E_, *H*_O_), *F*_IS_ values, average individual inbreeding coefficient (*I*) and pairwise *F*_ST_ values (Weir & Cockerham, 1984) were calculated. MICRO‐CHECKER v. 2.2.3 (Van Oosterhout et al., 2004) was used to check the data regarding genotyping errors and the presence of null alleles. The impact of null alleles on *F*_ST_ estimation was evaluated with FREENA (Chapuis & Estoup, 2007) using the excluding null alleles (ENA) method with 1000 bootstraps by comparing *F*_ST_ estimates before and after correction for null alleles.

We visualized the genetic structure by performing a discriminant analysis of principal components (DAPC) with the R‐package adegenet (Jombart, 2008) on individual and population level for bank voles and ticks.

For microsatellite data, we used STRUCTURE 2.3.4 software [32] to determine the number of genetic clusters (*K*). We tested the number of clusters from 1 to 15 with 10 iterations for each *K* (20 000 burn‐ins, 200.000 Markov chain Monte Carlo replicates in each run) using the “No admixture” model and assuming correlated allele frequencies to assess convergence of the probability ln P(X|K). R‐package *pophelper* (Francis, 2017) was used to determine the final number of clusters from Δ*K*, the rate of change in the log probability over all 10 iterations (Evanno et al., 2005), and to find the optimal individual alignments of replicated cluster analyses. The probability of each individual belonging to one of the *K* clusters got transformed into a three‐dimensional vector using principal coordinate analysis (PCoA) of the Euclidean distance of each cluster probability. The PCoA vectors were transferred via RGB algorithm to a genetic color code.

The presence of isolation by distance (IBD) was tested using Mantel's test between the genetic Euclidean distance of structure data and the geographic Euclidean distance among population sites. We tested for isolation by resistance (IBR) using Mantel's test between genetic Euclidean distance of structure data and least‐cost distance among population sites based on landscape features. Least‐cost distance was calculated using the R‐packages *terra* (Hijmans et al., 2022) and *gdistance* (van Etten, 2017).

IBD and IBR were computed with a Monte Carlo randomization test based on 999 replicates implemented in the R‐package *ade4* (Thioulouse et al., 1997).

TBEV isolate sequences were aligned using the R‐package *DECIPHER* (Wright, 2020). PCoA was calculated on the genetic Euclidean distance between the sequences, and principal coordinates were color‐coded via RGB transformation using the R‐package *dartR* (Mijangos et al., 2022). UPGMA tree was generated in MEGA Version 11 (Tamura et al., 2021) using Kimura 2‐parameter model and 500 replications.

We combined the results of the PCoA on population level for bank voles and ticks and on the isolate level for TBEV with geographical data in a synthesis map to illustrate the genetic constitution in space using ArcGIS Pro (ESRI, 2022).

### 2.5 Habitat suitability and corridor analysis

To determine the suitable habitats for bank voles and the connectivity of sampling sites within the sampling area, habitat suitability analysis and corridor analysis were conducted using the ArcGIS Toolbox “Spatial Analyst” (ESRI, 2022) and “Linkage mapper v 3.1.0” (McRae & Kavanagh, 2011). Landscape features (agriculture, vegetation, settlements, waterbodies, streams, traffic routes) were reclassified according to their suitability as bank vole habitats based on expert estimation. We modeled habitat suitability and suitable corridors for bank voles based on landscape features to detect possible landscape‐related barriers that could indicate a restriction in gene flow and habitat connectivity. Features like settlements, traffic routes, waterbodies, and streams were assigned a low habitat suitability value. Forests and hedges were characterized as highly suitable habitats. Agriculturally used areas, as well as moors and heaths, are assigned medium habitat suitability values. Based on the calculated habitat suitability value for each cell, an inverted resistance value for each cell was computed, too. The tools Linkage Pathway (McRae & Kavanagh, 2011) and Linkage Priority (Gallo & Greene, 2018) included in the GIS toolbox Linkage Mapper were used to carry out habitat connectivity analysis. Linkage pathway tool computed least‐cost paths representing the minimum cost‐weighted distance between each source and destination (Adriaensen et al., 2003). Linkage Priority tool weighs combinations of multiple factors regarding the sampling sites and linkages to quantify the relative conservation priority of each linkage, we used the default values to calculate linkage priority.

## 3 RESULTS

### 3.1 Abundance and demography of rodents and ticks

A total of 197 bank voles were caught on 15 sampling sites. The number of caught bank voles and their abundance varied strongly between sites, ranging from 3/1.6 on site II to 36/13.9 on site MM, with an average abundance of 7.1 bank voles per 100 trap nights, see Table 1.

The abundance of *Apodemus* spp. was evaluated too, since this genus is the bank voles' greatest competitor in forest habitats and their ecotones. Overall, 277 animals belonging to the genus *Apodemus* were identified with an average abundance of 9.8 per 100 trap nights. Number and abundance of *Apodemus* spp. ranged from 7/3.7 on plot II to 57/20.4 on plot DD. On five plots (AA, EE, JJ, MM, and NN), bank voles were the dominant species and showed higher abundances than *Apodemus* spp. On Plot FF, abundances were the same, whereas, on the remaining plots (BB, CC, DD, GG, HH, II, KK, LL, OO), *Apodemus* spp. were more abundant than bank voles.

Overall, 9% (24) of the examined bank voles tested TBEV‐positive were distributed on seven plots (AA, EE, GG, HH, JJ, LL, MM). Plots with bank voles being the dominant rodent showed the highest amount of TBEV‐positive bank voles except for plot NN, where no positive bank voles were caught. Only plots HH and LL showed TBEV‐positive bank voles, while *Apodemus* spp. being the dominant rodent species.

Three thousand six hundred twenty‐four ticks were sampled using the flagging method with cotton cloth on 15 sampling sites. Tick number and abundance per 100 m^2^ varied strongly between sites, ranging from 31/6.5 on site HH to 1425/129.2 on site MM. Sampled ticks comprised 55% Larvae, 37% Nymphs, 4% adult males, and 4% adult females. None of the sampled ticks tested positive for TBEV (see Table 1).

### 3.2 Genetic diversity of bank vole and tick populations

Sixty‐nine bank voles from Brandenburg et al. (2023) caught in 2019 on sites EE (*N* = 38) and MM (*N* = 31) were included in the genetic analysis, resulting in 266 bank voles that were genetically examined. The set of 12 microsatellite loci produced 139 alleles for the bank voles. The genetic diversity of all 15 populations is high, with significant differences between *H*_E_ and *H*_O_. *F*_IS_ and average inbreeding value (*I*) are comparable and in a low range. The populations with the lowest number of individuals sampled (CC, II) also show the lowest number of alleles per locus (*A*), the lowest observed and expected heterozygosity (*H*_E_, *H*_O_), and the lowest allelic richness (*A*_*R*_). No bank vole population shows any conspicuous features regarding the basic population genetic parameter.

Four hundred twenty‐eight ticks were genetically examined. The set of 12 microsatellite loci produced 123 alleles for the ticks. Within all populations, the level of genetic diversity is situated in the lower mid‐range, with significant differences between *H*_E_ and *H*_O_. *F*_IS_ and average inbreeding value (*I*) are comparable and in an intermediate range. No tick population shows any conspicuous features regarding the basic population genetic parameter. Table 2 gives an overview of all basic populations’ parameters for the 15 tick and and 15 bank vole populations.

**TABLE 2:** Microsatellite diversity indices of bank vole (*Myodes glareolus*) and tick (*Ixodes ricinus*) populations.

Plot ID	*Myodes glareolus*							*Ixodes ricinus*						
	*N*	*A*	*H*_O_	*H*_E_	*F*_IS_	*A*_R_	*I*	*N*	*A*	*H*_O_	*H*_E_	*F*_IS_	*A*_R_	*I*
AA	24	12	0.76	0.87	0.13	4.46	0.19	29	7.8	0.44	0.72	0.40	5.62	0.38
BB	5	6	0.75	0.84	0.13	4.23	0.20	30	7.6	0.46	0.72	0.37	5.82	0.37
CC	4	4	0.65	0.78	0.20	3.56	0.20	29	8.1	0.44	0.73	0.40	6.1	0.40
DD	10	9	0.73	0.86	0.17	4.46	0.21	29	9.0	0.48	0.75	0.37	6.4	0.37
EE	56	16	0.74	0.89	0.17	4.68	0.23	30	8.8	0.46	0.75	0.40	5.87	0.38
FF	12	9	0.71	0.87	0.19	4.47	0.21	29	8.1	0.41	0.74	0.46	5.86	0.42
GG	10	8	0.73	0.86	0.16	4.32	0.19	30	7.6	0.46	0.75	0.40	6.49	0.36
HH	16	10	0.69	0.86	0.20	4.42	0.24	28	8.9	0.40	0.75	0.48	6.12	0.43
II	3	4	0.67	0.83	0.23	4.08	0.17	29	8.1	0.41	0.76	0.47	5.93	0.43
JJ	16	10	0.76	0.86	0.12	4.41	0.18	29	7.9	0.43	0.76	0.44	5.69	0.40
KK	10	8	0.73	0.87	0.17	4.4	0.21	14	7.1	0.46	0.76	0.44	5.81	0.41
LL	13	10	0.76	0.87	0.13	4.45	0.18	29	7.8	0.41	0.71	0.45	6.51	0.43
MM	67	16	0.73	0.88	0.17	4.53	0.23	32	8.4	0.40	0.71	0.48	6.18	0.44
NN	8	8	0.69	0.86	0.21	4.4	0.23	32	9.2	0.41	0.77	0.42	6.33	0.40
OO	12	9	0.71	0.85	0.18	4.32	0.23	29	8.4	0.43	0.74	0.41	6.32	0.37
	*266*							*428*						

Abbreviations: *A*, the average number of alleles per locus; *A*_R_, mean allelic richness per population; *F*_IS_, *F*is‐Value; *H*_O_, observed and *H*_E_, expected heterozygosity; *I*, average inbreeding value; *N*, number of animals; Plot ID, plot identifier.

### 3.3 Genetic differentiation and genetic composition

#### 3.3.1 Bank voles

**FIGURE 1:**
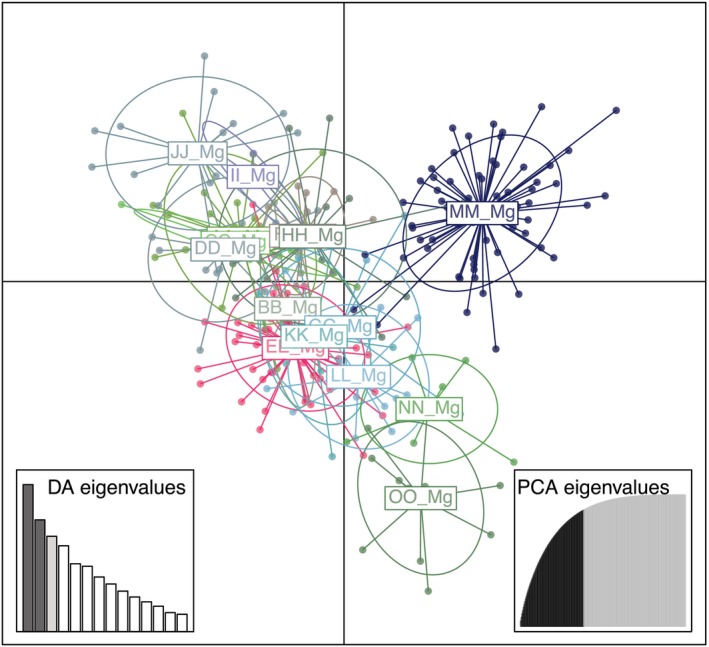
Result of DAPC analysis of 266 bank vole samples from 15 populations *color‐coded by* RedGreenBlue transformation.

MICRO‐CHECKER revealed signs of possible null alleles at three loci (CG16E2, CG12B9, CG15F7) across our dataset. To estimate the impact of possible null alleles, *F*_ST_ values were calculated using the ENA algorithm (FREENA), which corrects for null alleles. We observed minor differences between the corrected and uncorrected estimates of genetic differentiation, that do not seem substantial (overall *F*_ST_ using ENA, *F*_ST_ = 0.037003; without ENA, *F*_ST_ = 0.038558). Both calculated *F*_ST_ values deviate significantly from zero (*p* < .05). DAPC analysis based on allele composition shows obvious genetic differentiation between the 15 bank vole populations (Figure 1).

**FIGURE 2:**
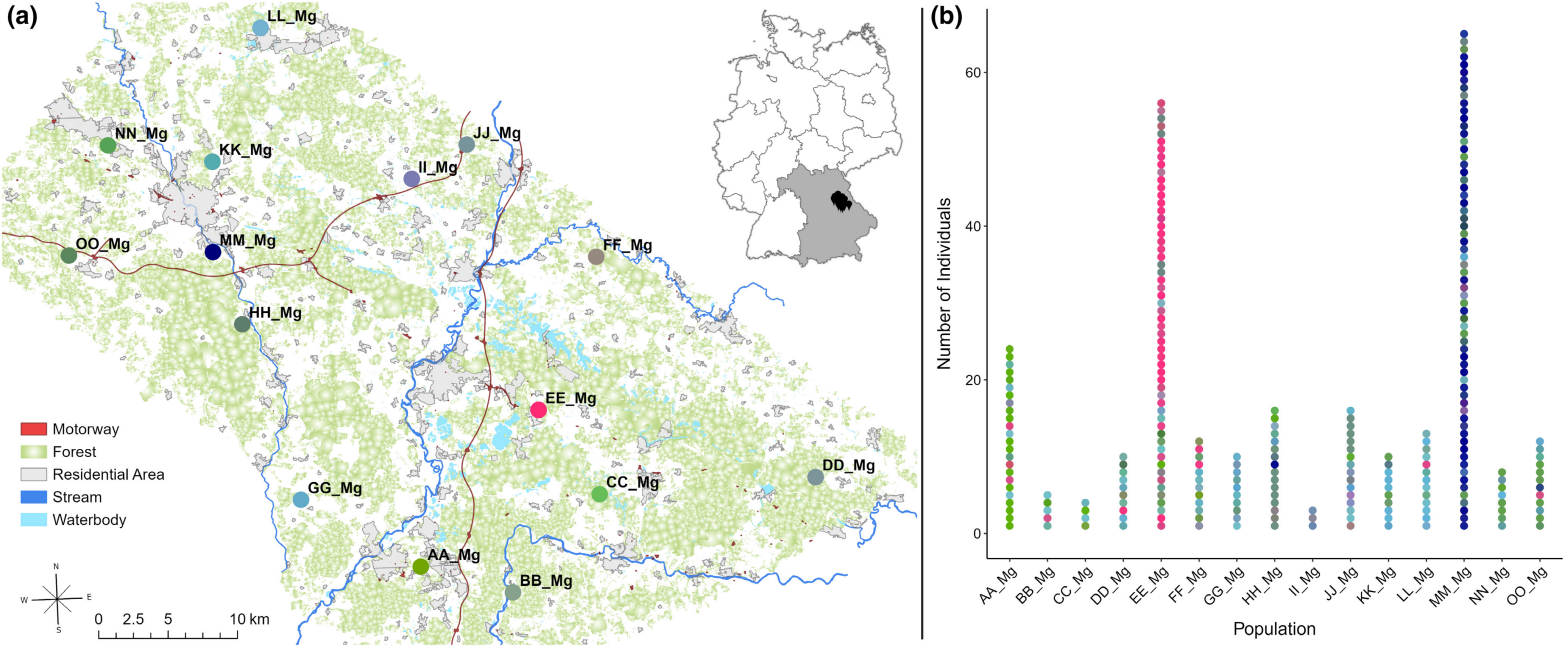
Genetic differentiation and composition of bank vole populations. (a) Synthesis map combining geographical and genetic data of the bank vole populations after PCoA analysis based on the genetic cluster data, visualized by RedGreenBlue transformation (b) Bank vole individuals cluster affiliation based on PCoA analysis visualized by RedGreenBlue transformation.

PCoA analysis and RedGreenBlue transformation of the probability of each bank vole belonging to one of eight clusters show that particularly populations MM and EE hold a special position among the 15 bank vole populations. The results of PCoA analysis and RGB transformation in conjunction with geographical data are shown in Figure 2a. Similar colors represent similar genetic constitutions of individuals or populations. Figure 2a suggests comparatively high inter‐population genetic variation, with, especially populations EE and MM differing strongly from each other but also from all other populations. Figure 2b shows that intra‐population genetic variation is low.

Mantel's test revealed that the geographic distance, respectively the landscape resistance between sampling sites, are not correlated with the genetic Euclidean distance of bank voles (IBD: simulated *p*‐value = .200; IBR: simulated *p*‐value = .432). Therefore, the genetic differentiation of bank vole populations can not only be explained by geographical distance or landscape resistance between the sampling sites. The outstanding genetic compositions of bank voles at sites EE and MM could contribute to that result, emphasizing their special position among the 15 populations.

#### 3.3.2 Ticks

**FIGURE 3:**
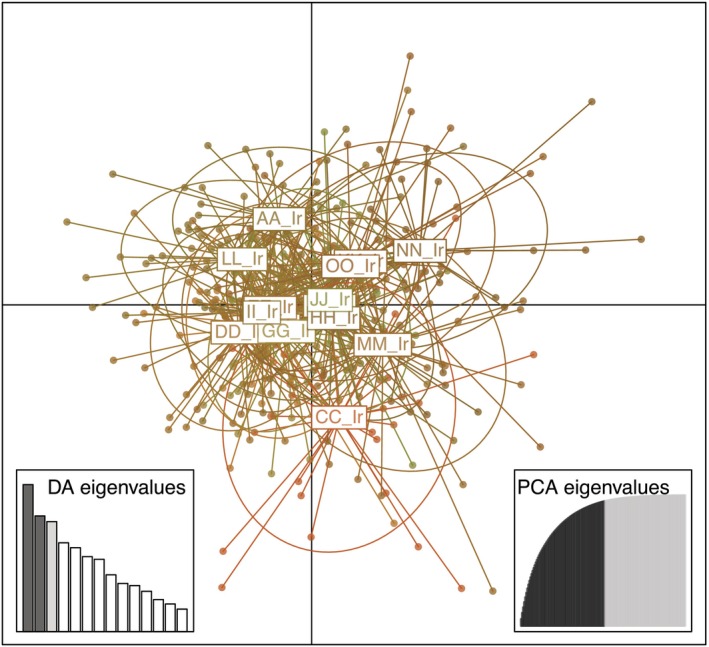
Result of DAPC analysis of 428 *Ixodes ricinus* samples from 15 populations color‐coded by RedGreenBlue transformation.

The 15 tick populations show very low genetic differentiation and population structure. MICRO‐CHECKER revealed signs of possible null alleles at 10 loci across our dataset loci IRic05 and IRic08 did not show signs of null alleles. To estimate the impact of possible null alleles, *F*_ST_ values were calculated using the ENA algorithm (FREENA), which corrects for null alleles. We observed some differences between the corrected and uncorrected estimates of genetic differentiation, that do not seem substantial (overall *F*_ST_ using ENA, *F*_ST_ = 0.004958; without ENA, *F*_ST_ = 0.002914). Both calculated *F*_ST_ values deviate significantly from zero (*p* < .05). DAPC analysis based on allele composition shows little genetic differentiation between the 15 tick populations (Figure 3).

**FIGURE 4:**
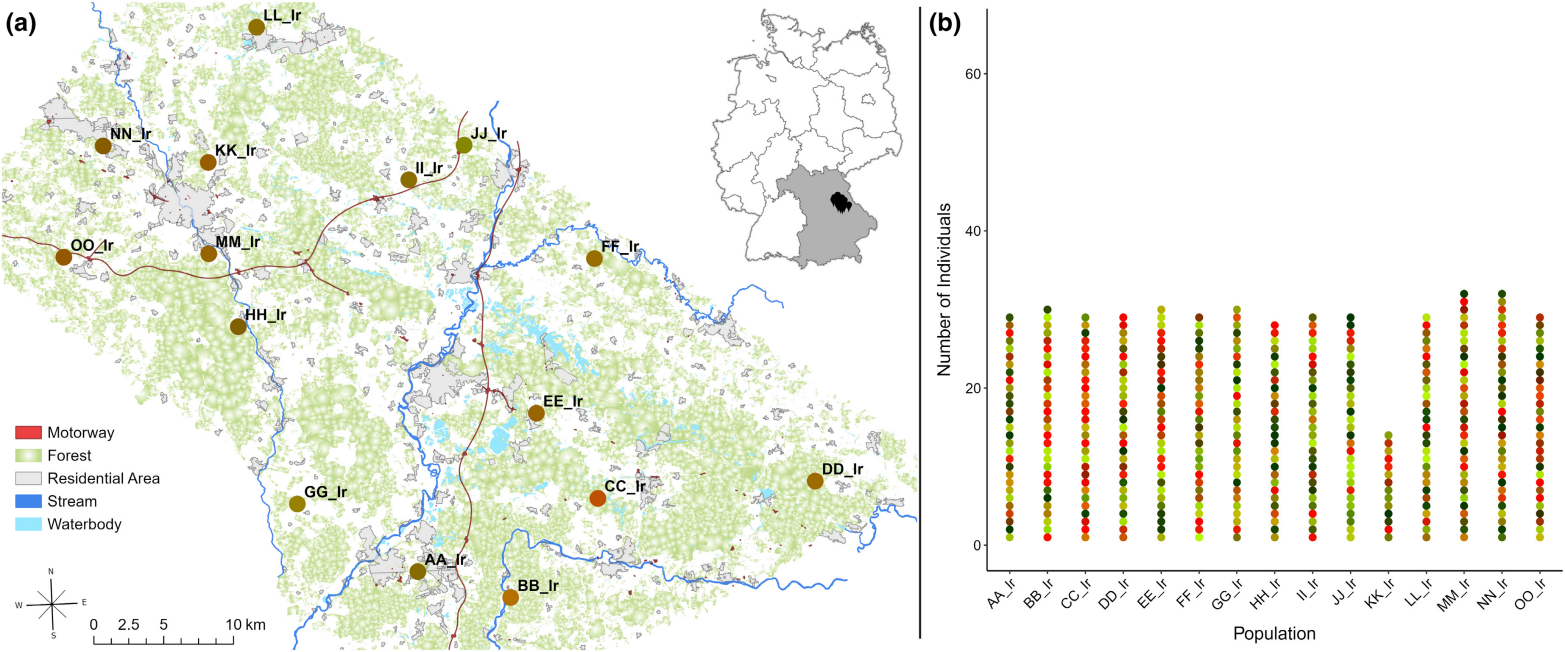
Genetic differentiation and composition of *Ixodes ricinus* populations. (a) Synthesis map combining geographical with genetic data of the *I. ricinus* populations after PCoA analysis based on the genetic cluster data, visualized by RedGreenBlue transformation (b) *I. ricinus* individuals cluster affiliation based on PCoA analysis visualized by RedGreenBlue transformation.

PCoA analysis and RedGreenBlue transformation of the probability of each tick pool belonging to one of three clusters also show very few genetic differentiations between the populations, with no populations standing out (Figure 4). These results illustrate the very similar genetic constitution between populations (Figure 4a) with high individual differentiation within the populations (Figure 4b). The results show a very mixed genetic pattern without any genetic structures over populations or groups of populations. This indicates a strong gene flow between the tick populations.

#### 3.3.3 TBE virus

E‐Gene sequences were successfully generated based on TBEV isolates derived from six plots (AA, EE, II, KK, LL, MM) within the study area (*N* = 15 plots).

**FIGURE 5:**
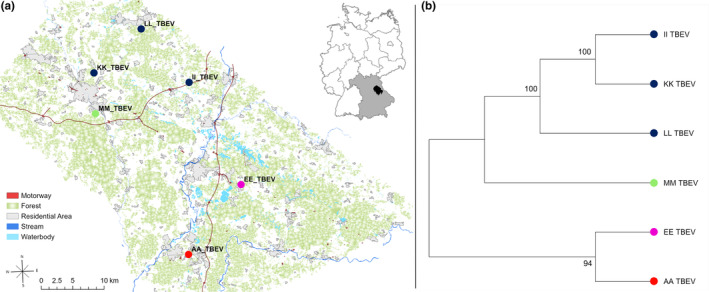
Genetic differentiation of TBE virus isolates. (a) Synthesis map combining geographical with genetic data of the TBEV isolates after PCoA analysis visualized by RedGreenBlue transformation (b) UPGMA Tree of TBEV virus isolates based on PCoA analysis, nodes are color‐coded according to the RedGreenBlue transformed PCoA axis. This phylogeny is based on 46 silent point mutations.

We could detect strong genetic differentiation (see Figure 5). The UPGMA tree in Figure 5b indicates a genetic north/south separation of TBEV. In the north, the TBEV isolate MM differs strongly from the other three isolates (KK, II, LL). In the south, TBEV isolate EE also differs from AA.

### 3.4 Habitat suitability and corridor analysis

**FIGURE 6:**
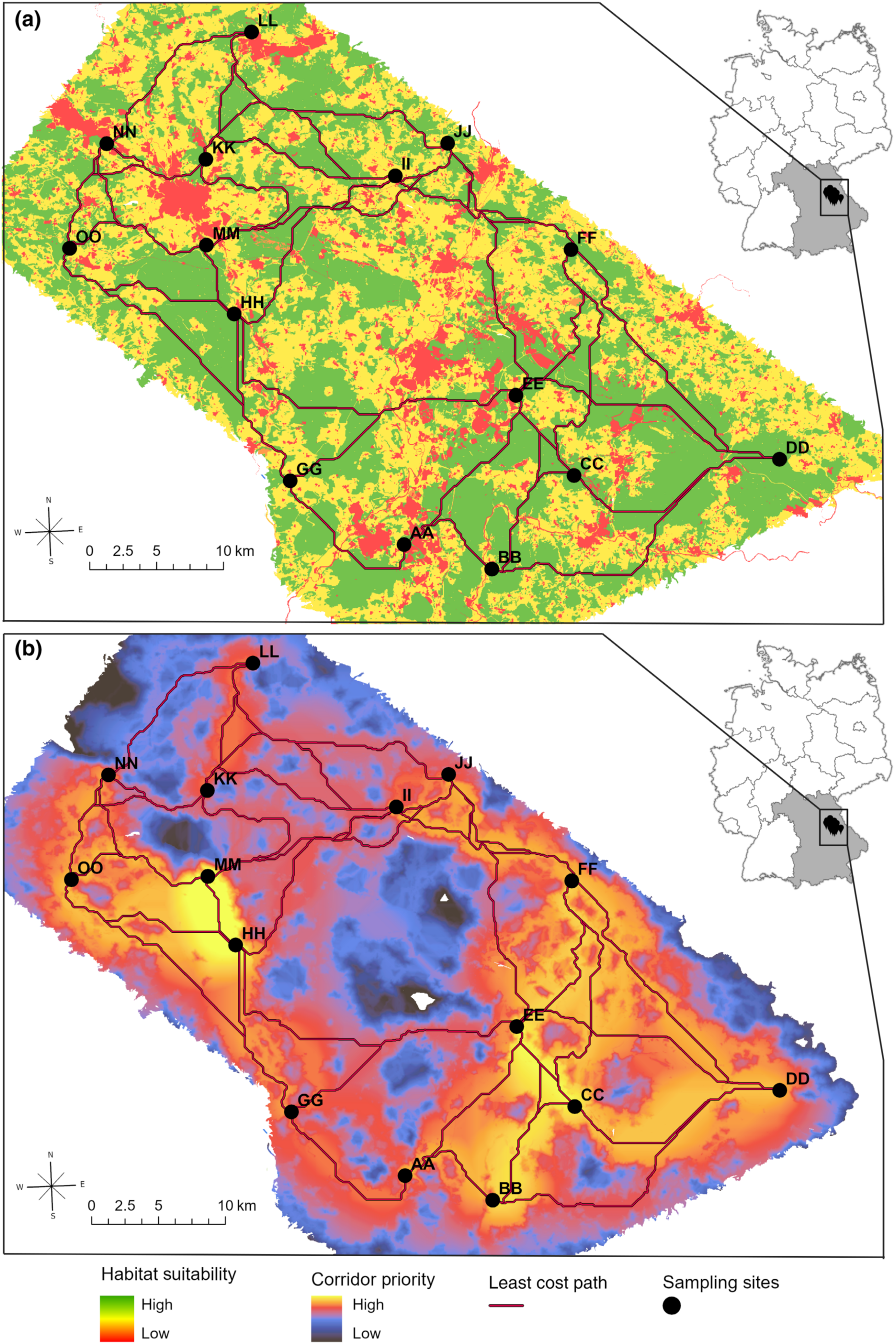
Habitat suitability and corridor analysis for bank voles. (a) Habitat suitability surface of the sampling area (b) Modeled least‐cost paths and corridors for bank voles connecting the sampling sites.

The landscape in the study area is very heterogeneous (Figure 6a). The biggest settlements are Amberg, Sulzbach‐Rosenberg, Hirschau in the north and Schwandorf, Schwarzenfeld, and Burglendenfeld in the south (see larger red patches in Figure 6a). Multiple waterbodies, streams, and lakes are situated in the south of the study area. Moreover, two main motorways cross the area (A6: west/north east and A93: north/south). Relatively large, connected forest areas are found around the settlements, for example, in the west, the southeast, and the north of the sampling area.

Figure 6b shows the least‐cost paths between all sampling sites. In the background, corridor priority is displayed. Large settlements show highest resistance. The most important corridors for bank voles are in the southeast, including sampling sites AA, BB, CC, DD, EE, FF, II, and JJ, and in the northwest, including sampling sites HH, MM, NN, and OO.

## 4 DISCUSSION

TBEV is circulating in nature in a transmission cycle, which is generally accepted to occur between the vector (ticks) and the host (small mammals). While the importance of the ticks is obvious in maintaining this natural TBEV focus, the biological role of the hosts is less clear. Even the role of the particular mammal species is under discussion. While some researchers prefer the main role to mammals of the genus *Apodemus* (family Muridae), often mainly bank voles (family Cricetidae) are found positive in natural TBEV foci (Brandenburg et al., 2023; Esser et al., 2022). One mystery of the TBEV transmission still is its focality on so‐called microfoci or natural foci (Borde et al., 2022).

However, to the best of our knowledge, no genetic analyses, neither of the vectors nor of the natural hosts of TBEV in a well‐defined natural microfocus of TBEV, have been conducted so far. Therefore, the impact of the genetic composition of vector or host populations on the development and maintenance of TBEV microfoci is unclear.

### 4.1 Bank voles

Our findings of 9% TBEV‐positive bank voles over the sampling period are in accordance with the findings of Brandenburg et al. (2023) in the same research area. Zöldi et al. (2015) detected up to 20% of seroprevalence in bank voles in Hungary. Grzybek et al. (2018) found seroprevalence rates of TBEV of about 14.8% in Poland, with significant variations between years and sampling sites.

The high degree of genetic diversity of bank voles corresponds with the findings based on comparable microsatellite marker analysis done by Gerlach and Musolf (2000) in the same species in the southwest of Germany and Switzerland and with the findings of Redeker et al. (2006) in Denmark. Populations CC and II, which show a lower degree of genetic diversity, must be taken with precaution due to their low sample sizes.

The degree of habitat fragmentation and the number of corridors connecting habitats are important determinants of migration ability and gene flow (Aars & Ims, 1999; Delaney et al., 2010). Guivier et al. (2011) found a high genetic homogeneity between populations in extended, mostly connected woodlands. This corresponds with our findings to a certain degree. The habitat connectivity model detected suitable paths and corridors between all sampling sites providing habitat connectivity, and we found similar genetic structure and differentiation of all 15 bank vole populations, excluding populations EE and MM. This underlines the special character these two populations hold among the 15 sampling sites. The overall populations low individual inbreeding values, high heterozygosity values, the small differences between observed and expected heterozygosity, and the lack of significant correlation between genetic distance and geographical distance, respectively, landscape‐related resistance indicate that the genetic differentiation of the bank vole populations is not only determined by landscape or drift effects. In a different rodent species, Saxenhofer et al. (2019) have shown, that host (common vole, *Microtus arvalis*) and pathogen (Tula orthohantavirus (TULV)) genetics are linked. They found genetically different TULV in a geographical region, where common voles of two distinct evolutionary lineages interact and interbreed. Underlining the fact, that pathogens can drive host evolution.

### 4.2 Ticks

A study in a similar region of Germany tested 8805 ticks for TBEV via RT‐PCR and discovered a TBEV prevalence, evaluated as the minimum infection rate (MIR), of 0.26% (Zubriková et al., 2020). Ott et al. (2020) analyzed 17,893 ticks and found comparatively low MIRs of 0.4% in a TBE high‐risk endemic area in southwestern Germany. With MIR being this low in the questioned area, our comparably low sample size (3624) could account for the fact that no TBEV‐positive tick was detected in our study.

The 15 *Ixodes ricinus* populations’ genetic divergence is very low, the populations do not differentiate based on the genetic constitution, dominant genetic clusters, or structure based on geographical distance. A nearly panmictic population of *I. ricinus* is to be assumed in the researched area. Comparative research on *I. ricinus* populations’ genetics to capture spatial population structure on large geographical scales commensurate with our findings and state that *I. ricinus* only show genetic structure and deviation from panmixia at larger geographical scale than our research area covers (Meeüs et al., 2002; Noureddine et al., 2011; Poli et al., 2020).

These findings can be explained by the fact that *I. ricinus's* live cycle includes three hemophagic stages (Medlock et al., 2013) and very low host specificity, resulting in *I. ricinus* having been recorded from over 300 terrestrial vertebrate species (Gern & Humair, 2002, Gray et al., 2021), including birds, reptiles, small and large mammals with respectively large ranges.

Climate, weather, and vegetation influence the survival of ticks in certain habitats, but due to their very low host specificity combined with their very limited ability to spatially migrate on their own, ticks do not actively contribute to their location of habitat (Gray et al., 2021). Therefore, modeling habitat suitability for ticks regarding its interrelation with TBEV transmission and TBEV focus dispersal would not add any value to the interpretation of the results.

### 4.3 Association of host and vector's population structure with TBEV

Bank voles and ticks show very unequal genetic differentiation patterns. Tick populations do not show any genetic structure throughout the study area. This underlines the little to no impact any biological or anthropogenic barriers have on *Ixodes ricinus* genetic diversity and differentiation, at this spatial scale. Our results suggest that ticks' genetic features do not contribute to sustain long‐term focalitiy of TBEV. In contrast, bank voles play an important role in the sustainability of long‐term TBEV foci.

Bank vole populations differ strongly genetically between sampling sites. Especially populations EE and MM show very different genetic compositions compared with the remaining populations and each other. According to our habitat suitability and connectivity model, the genetic differentiation of sampling sites EE and MM cannot be explained by habitat suitability or any landscape‐related barrier restricting habitat connectivity and gene flow. This indicates that factors other than geographical distance and landscape‐related resistance contribute to the different genetic compositions of bank vole populations in these two long‐time monitored TBEV foci. Nonsignificant tests on IBD and IBR underline this assumption. TBEV clusters into four genetic groups. Two groups are located in the north, and two groups are located in the south of the study area, with sampling sites EE and MM differing from each other and from the remaining populations in their region. Even though our sample size regarding TBEV isolates is comparably small, our results coincide with the characterization of multiple TBEV strains by Weidmann et al. (2013) (AA = Burglengenfeld, EE = Heselbach, MM = Haselmühl).

On multiple sites, TBEV‐positive bank voles were detected by serology and RT‐PCR but only bank vole populations on sites EE and MM show extraordinary genetic constitutions. The same accounts for the genetic analysis of TBEV isolates, where EE and MM also differ genetically from their surrounding sites.

The major difference between sites EE and MM and all other sites where TBEV was detected (AA, GG, HH, JJ, LL) is the fact that these plots are long‐term (>10 years) established TBEV foci. Therefore, we infer that the genetic constitution of bank voles and the establishment and maintenance of natural TBEV foci seem to be associated.

This leads to the assumption that bank voles do not simply serve as a TBEV reservoir, but their genetic composition also influences the establishment and maintenance of long‐term natural TBEV foci. However, this interrelation needs to be investigated further in terms of how and to which extent TBEV and *M. glareolus* genetics are associated.

## AUTHOR CONTRIBUTIONS

**Lea Kauer:** Data curation (lead); formal analysis (lead); investigation (equal); methodology (equal); software (equal); visualization (lead); writing – original draft (lead). **Gerhard Dobler:** Conceptualization (equal); data curation (equal); funding acquisition (equal); investigation (equal); resources (equal); writing – review and editing (equal). **Hannah M. Schmuck:** Data curation (equal); investigation (equal); resources (equal); writing – review and editing (equal). **Lidia Chitimia‐Dobler:** Data curation (equal); investigation (equal); writing – review and editing (equal). **Martin Pfeffer:** Conceptualization (equal); data curation (equal); funding acquisition (equal); resources (equal); writing – review and editing (equal). **Ralph Kühn:** Conceptualization (equal); data curation (equal); formal analysis (equal); funding acquisition (equal); methodology (equal); project administration (equal); resources (equal); software (equal); supervision (equal); validation (equal); writing – original draft (equal); writing – review and editing (equal).

## CONFLICT OF INTEREST STATEMENT

The authors declare that there are no conflicts of interest.

## Supporting information


Appendix S1

## Data Availability

The data, including metadata that support the findings of this study, are openly available in Dryad at https://doi.org/10.5061/dryad.cnp5hqcbn. (temporary link while the manuscript is in peer review and the dataset is unpublished: https://datadryad.org/stash/share/zOiwMLYNF60MjdAFg3IJoM8cy9mwgzQQoQ7AEqkVY0g).
