# Supplementary material for: Interrelation of the spatial and genetic structure of tick‐borne encephalitis virus, its reservoir host (Myodes glareolus), and its vector (Ixodes ricinus) in a natural focus area
Source: Ecol Evol. 2024 Aug 19;14(8):e70163. doi: 10.1002/ece3.70163 (PMC11333545; doi:10.1002/ece3.70163)
Supplement: Supplementary file 1 — Appendix S1 [file ECE3-14-e70163-s001.docx]

# Appendix

| ***Myodes glaroelus*** | | | | |
| --- | --- | --- | --- | --- |
| Multiplex PCR | Locus | Primer Sequence (F: 5´- 3´)(R:3´- 5´) | Dye | Primer concentration, μM |
| Mg_Mix I | CG13G2 | F: ACGACTTAGAGGGCTAGTTG | 6-FAM | 0.15 |
|  |  | R: GGAGTGACACAGGAGTTTGT |  | 0.15 |
|  | CG5F6 | F: CCTAGCCTAACTCAGGAAAGT | 6-FAM | 0.2 |
|  |  | R: GAAGTCTGCATTGTCAAACA |  | 0.2 |
|  | CG16E2 | F: CTTATTGACCCCACCTACCT | HEX | 0.2 |
|  |  | R: AATAAAGCCAGGCTGGAATA |  | 0.2 |
|  | CG17E9 | F: AATACTTCCAGTGCTGATGC | TAMRA | 0.2 |
|  |  | R: TCAGAACCCTGTTCTCTGAC |  | 0.2 |
|  | CG7C9 | F: TTTCTCTGGGACTAAACAGC | TAMRA | 0.25 |
|  |  | R: TGTGCATGTCAGTTTCCTTA |  | 0.25 |
| Mg_Mix II | CG15F7 | F: ATATTTCCCTGAGGGTGAAC | 6-FAM | 0.15 |
|  |  | R: GGCTGAGAGATACATTATGGTC |  | 0.15 |
|  | CG12B9 | F: AGCTGGGGTTACACAGAGA | 6-FAM | 0.2 |
|  |  | R: GTAGTACATGGAAGACAAGG |  | 0.2 |
|  | CG13F9 | F: TGCTCACACAAACTGTGATT | HEX | 0.2 |
|  |  | R: TAACCGGGGAGTAGAGAAA |  | 0.2 |
|  | CG5G6 | F: CAGCAACCACATAGTGACTC | HEX | 0.2 |
|  |  | R: CCTCAGATTTCTCCTTCACA |  | 0.2 |
|  | CG12A7 | F: TCTAGGCAAGTCGAGAGTGT | HEX | 0.25 |
|  |  | R: GGCTTTCTCCAAGACTTTCT |  | 0.25 |
|  | MSCg-15 | F: CTACTGAGTTCCCGTGGTAG | TAMRA | 0.25 |
|  |  | R: TGTGAATTTGCATCAAGAGA |  | 0.25 |
|  | CG16E5 | F: AATGACAACTCTTTCCCTGTC | TAMRA | 0.25 |
|  |  | R: TTATTAGAAGGTCAGGGAACA |  | 0.25 |

| ***Ixodes ricinus*** | | | | |
| --- | --- | --- | --- | --- |
| Multiplex PCR Mix | Locus | Primer Sequence (F: 5´- 3´)(R:3´- 5´) | Dye | Primer concentration, μM |
| Ir_Mix I | IR27 | F: CATCGCTAGTGGCTAGAG | TAMRA | 0.09 |
|  |  | R: TTATAACCCGAGGTCGTAAAA |  | 0.09 |
|  | IR32 | F: TCGACAAGTGCAGTGGAGAC | HEX | 0.12 |
|  |  | R: GTTTCCTACCACAGATTCTCC |  | 0.12 |
|  | IR39 | F: ATACCCGTAGAACGAGAG | HEX | 0.06 |
|  |  | R: GTTTTTCAAGATTTCCGCC |  | 0.06 |
|  | IR8* | F: AGTCCATATGCTTATATACCCG | TAMRA | 0.21 |
|  |  | R: CTGTATCTACAACCGGTCGT |  | 0.21 |
|  | IRic09 | F: AAAAGACCCCAGAAACAA | 6-FAM | 0.18 |
|  |  | R: GGGGAAGAAAATATGCTAA |  | 0.18 |
|  | IRN-7 | F: CGGATGATCAATAGTCGATTCC | 6-FAM | 0.12 |
|  |  | R: CCTAGTCACAAACTCTACCAAGTTA |  | 0.12 |
| Ir_Mix II | IRic05 | F: CCTTACCAACCCTGTGTC | HEX | 0.09 |
|  |  | R: GAGCCGAATTTTATGCAC |  | 0.09 |
|  | IRic08 | F: TCATTGTCCCTTCCAGTACG | TAMRA | 0.12 |
|  |  | R: AGAAAATAAGCGCCGAGAAA |  | 0.12 |
|  | IRic11 | F: AGCTACGAGACTACATCAAAA | HEX | 0.12 |
|  |  | R: TCAAAGACAGTGACGCTTA |  | 0.12 |
|  | IRic13 | F: AATGACGCCAGCGAGATAAT | HEX | 0.12 |
|  |  | R: TCTATATAGGGGGTGGCGAAT |  | 0.12 |
|  | IRN-14 | F: CGTAATTGCGCCTTGTTGA | TAMRA | 0.21 |
|  |  | R: GAGCGCAAATTGAAGTGAGC |  | 0.21 |
|  | IRN-3 | F: TTTGGGGGAGTGACAGAAAC | 6-FAM | 0.12 |
|  |  | R: AAAACAAATGCCCAAACACC |  | 0.12 |
|  | * X-linked locus | |  |  |
